# Chemistry, pharmacology and analysis of *Pseudostellaria heterophylla*: a mini-review

**DOI:** 10.1186/s13020-019-0243-z

**Published:** 2019-05-23

**Authors:** De-jun Hu, Farid Shakerian, Jing Zhao, Shao-Ping Li

**Affiliations:** 1 State Key Laboratory of Quality Research in Chinese Medicine, Institute of Chinese Medical Sciences, University of Macau, Macau, China; 2 College of Pharmacy, Chengdu University of Chinese Medicine, Chengdu, China

**Keywords:** *Pseudostellaria heterophylla*, Chemistry, Pharmacology, Analysis

## Abstract

*Pseudostellaria heterophylla* is one of the well-known traditional Chinese medicines and has been used in clinics for 100 years in China. The chemistry and pharmacology of *P. heterophylla* were reviewed to understand its active compounds. Then analysis of these compounds related to quality control of this herb was discussed. For the analysis of chemicals, three aspects have been discussed in this review. The first two aspects focused on the methodologies for analysis of cyclic peptides and carbohydrates in *P. heterophylla*, respectively. The last one dealt with the other methods used for identification of *P. heterophylla.* Some rich chemicals such as oligosaccharides in this plant were rarely evaluated. Many analyses were performed on this plant, however, few of them were accepted as quality control method.

## Introduction

*Pseudostellaria heterophylla*, *tai*-*zi*-*shen* (太子参) or *hai*-*er*-*shen* (孩儿参) in Chinese, is a well-known traditional Chinese medicines (TCMs) first officially recorded in *Ben Cao Cong Xin*, which contains 721 kinds of herbs, by Wu Yiluo in 1757 [1]. *P. heterophylla* was considered as one of the precious medical material from ancient China and now is one of the most commonly used TCMs in clinic, which invigorating spleen, replenishing qi, moistening lung and benefiting blood. It has been used for treatment of fatigue, spleen asthenia, anorexia, asthenia after severe illness and cough due to lung dryness [2–5]. This medicine is often used for children as a substitute of ginseng because of its mild effects [6].

*Pseudostellaria heterophylla* mainly distributed in Liaoning, Hebei, Shandong, Anhui and Sichuan provinces. Ningde (Fujian Province) and Shibing (Guizhou Province) in China offer the most suitable envionment for *P. heterophylla* cultivation [7]. However, consecutive monoculture of this plant will lead to a serious decline of biomass and quality of its underground tubers. Farms used for cultivation of *P. heterophylla* can only be replanted once every 4 years [8, 9]. As the sources of wild *P. heterophylla* with high quality in geo-authentic production zone are limited and the demand for this medicinal material is rising annually, the government has established a large-scale cultivation areas for it in Jurong (Jiangsu Province), Zherong (Fujian Province), Shibing (Guizhou Province) and Xuancheng (Anhui Province) of China [10]. However, due to differences of ecological environments, accumulation of active components in wild and cultivated *P. heterophylla* and their quality have shown significant differences [11, 12]. Therefore, it is necessary to understand chemical components and pharmacological activities of *P. heterophylla* before establishing an effective quality control method to ensure its safety and efficacy [13].

## Chemical constituents in *P. heterophylla*

**Fig. 1:**
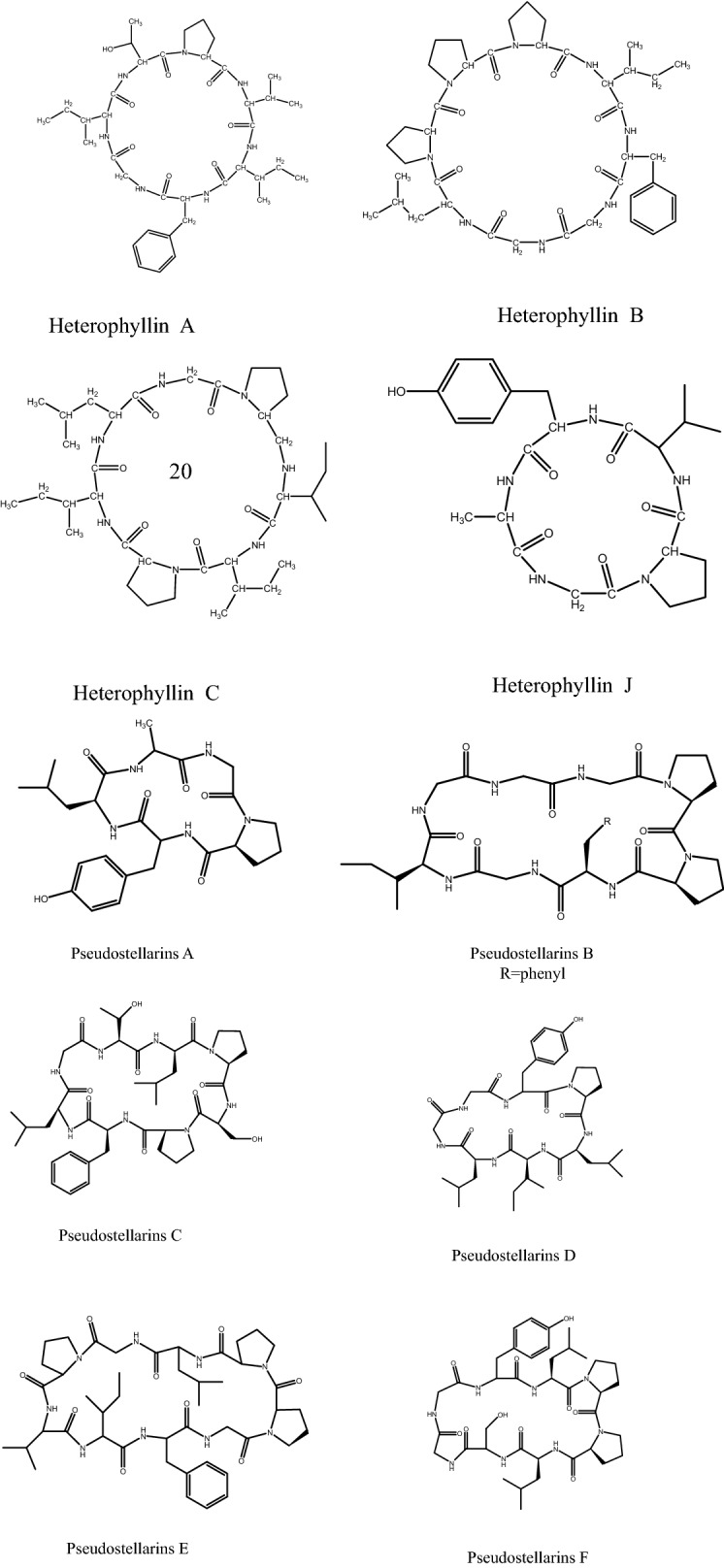

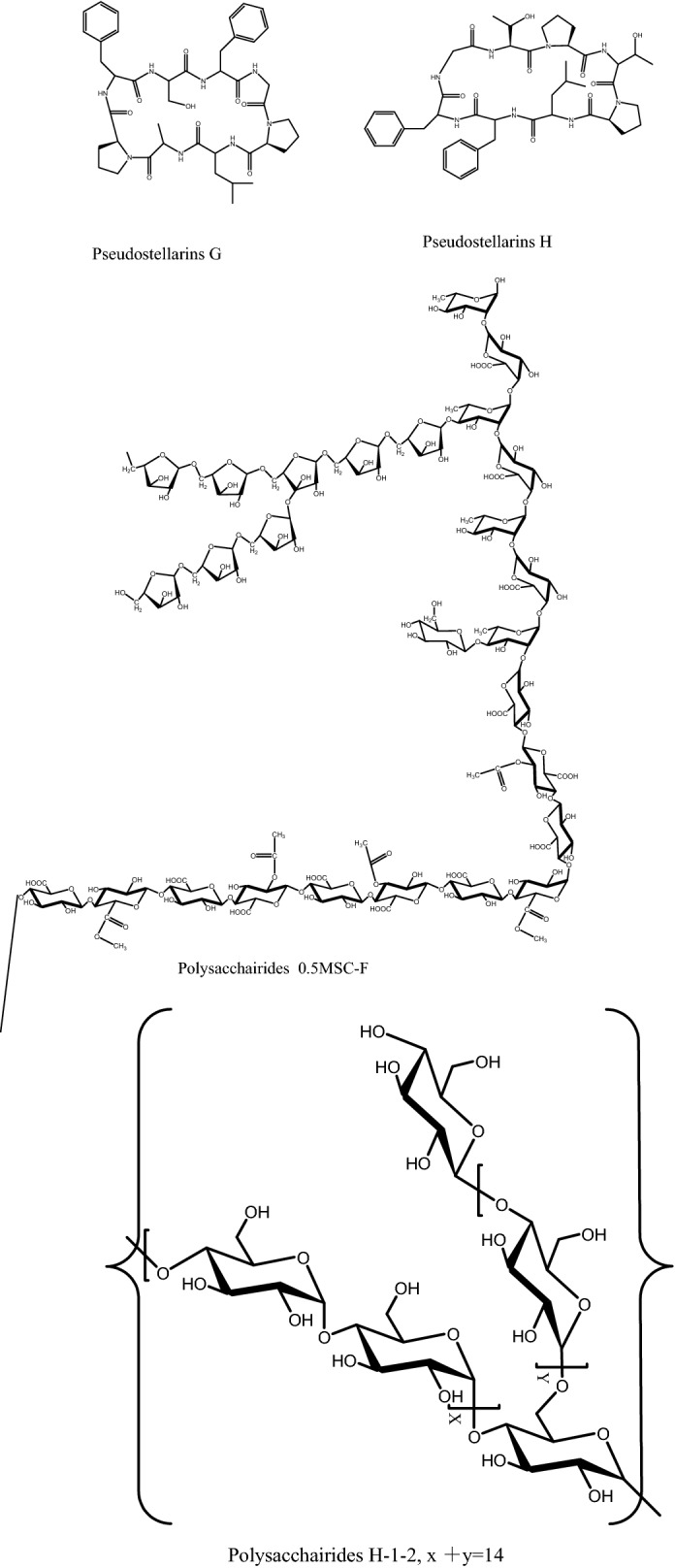
Structures of main chemicals in *Pseudostellaria heterophylla*

Various components were found in *P. heterophylla*, including cyclic peptides (pseudostellarin), polysaccharides, amino acids, saponins, and sapogenins based on chemical studies [14]. In recent years, cyclic peptides with special structures (Fig. 1) isolated from *P. heterophylla* have attracted many researchers’ interest. And high-speed counter-current chromatography (HSCCC) was demonstrated to be an efficient separation method for cyclic peptides [15–17]. Up to date, pseudostellarin A-G have been separated from *P. heterophylla* [18–21], which was summarized in Table 1. In addition, polysaccharides (Fig. 1), as one of main bioactive components in *P. heterophylla*, have been reported to exhibit multiple pharmacological activities [22]. Lectins with high hemagglutination activity were also found in *P. heterophylla* [23, 24]. They also have minor inhibitory effect on glycohydrolases, such as α-glucosidase, β-glucosidase and β-glucuronidase which are involved in HIV infection [24]. However, these lectins were devoid of antifungal activity, labile to acid and alkali and also exhibited poor thermostability [23].

**Table 1:** Main chemical constitutes in *P. heterophylla*

Type of components	Components purified	References
Peptides	Heterophyllin A, B, D, J	[15, 17, 19, 61–64]
	Pseudostellarins A–C	[20, 63]
	Pseudostellarins D–F	[21, 65]
	Pseudostellarin G	[18]
	Pseudostellarin H	[66]
Polysaccharide	Rhamnogalacturonan I	[26]
	H-1-2 (MW 1.4 × 104 Da, a type of glucan, main chain with 1 → 4 linked glucose, and a small amount of branched chain with 1,6-linked glucose)	[27]
	PH-I A, PH-I B and PH-I C	[22]
Lectin	36 kDa lectin	[23, 24]

## Pharmaceutical activities of *P. heterophylla*

Based on the abundant chemical constituents, *P. heterophylla* has multiple pharmaceutical activities including immunomodulatory [3, 25], antidiabetic [26–29], antitussive [5], antioxidant [30] activities, as well as protective effects on retinal injury and exercise-induced oxidative stress etc. [31–33].

Plant cyclopeptides comprise a large group of small molecules from natural medicines, which exhibit various pharmacological activities, such as immunomodulatory, anti-inflammatory, antioxidant, anti-aging and antitumor effects [34, 35]. Previous studies showed that heterophyllin B, one of main cyclopeptides in *P. heterophylla*, effectively suppressed the adhesion and invasion of human esophageal carcinoma cells by mediating PI3 K/AKT/β-catenin pathways and regulated the expression levels of adhesion- and invasion- associated genes [36]. Furthermore, cyclopeptides have been demonstrated as the major active components correlated to the cytotoxic activities against three human tumor cell lines (MGC80-3, HepG2 and RKO) [37].

In recent years, increasing studies have been focused on the bioactivities of polysaccharides from *P. heterophylla.* The fraction riched with polysaccharides of *P. heterophylla* has protective effects against cobalt chloride-induced hypoxic injury in H9c2 cell [14]. Crude polysaccharides from *P. heterophylla* also can improve exercise endurance and have protective effects against oxidative stress [31–33]. Polysaccharides with molecular weight of 50 kDa - 210 kDa are not only significantly lowering blood sugar but also reducing total triglyceride level in serum [28]. Polysaccharides of *P. heterophylla* have been proved their benefits to chronic fatigue syndrome. That may be why *P. heterophylla* is usually used as a tonic herb [38]. However, crude polysaccharides from *P. heterophylla* are commonly used. A water-soluble, pectic polysaccharide with molecular weight of 48 kDa, composed of rhamnose, galactose, arabinose and galacturonic acid and 1,4-linked galacturonic acid as main chain with small amount of 1,2-linked rhamnose, could obviously stimulated insulin secretion [26]. A novel homogeneous polysaccharide, named as H-1-2, was also isolated from *P. heterophylla* polysaccharide. The mean molecular weight of H-1-2 was 14 kDa and it was only composed of d-glucose monosaccharide. In vitro, HepG2, 3T3-L1, and L6 cells were used to assess cellular glucose consumption and cellular glucose uptake. The results showed that H-1-2 could clearly increase glucose uptake and utilization in muscle and adipose cells, which is beneficial for screening leading compounds of anti-diabetes [27]. The saponins extract from *P. heterophylla* has also been demonstrated to have protective effects on retinal laser injuries [4]. In addition, ethyl acetate fraction extracted from *P. heterophylla* exhibited a dose-dependent antitussive effect [5].

## Chemical analysis of *P. heterophylla*

Various methods have been developed to analyze the components in *P. heterophylla*. High performance liquid chromatography (HPLC), thin layer chromatography (TLC), gas chromatography (GC), matrix-assisted laser desorption/ionization mass spectrometry (MALDI-MS), near infrared (NIR) spectroscopy and nuclear magnetic resonance (NMR) etc. have been applied for characterization of components in *P. heterophylla*, which were summarized in Table 2. Peng et al. evaluated the concentration of heavy metals in cultivation soils and *P. heterophylla,* and their bioconcentration factors (BFs) of investigated heavy metals are not higher than 0.5 except for Cd, where Pb and As were especially low. Only Cd could be enriched slightly in *P. heterophylla* while others could not [39].

**Table 2:** Chemical analysis of *P. heterophylla*

Analytes	Methods	References
Pseudostellarin A, C, D, and G	HPLC–ESI–MSn	[25]
Pseudostellarin A, B, C, D, E, G	HPLC-APCI (atmospheric pressure chemical ionization)-MS	[67]
Pseudostellarin A, B, E, F, G, Heterophyllin A, B, D	UPLC-triple TOF–MS/MS; UPLC-ESI-TOF MS/MS	[64, 68, 69]
Maltotriose, sucrose, thyronine, inosine triphosphate, pseudostellarin A, B, D, F, heterophyllin A and sphinganine etc.	UPLC-triple TOF–MS/MS	[44–46]
21 compounds	Ultra-performance liquid chromatography-triple time-of-flight mass/mass spectrometry (UPLC-triple TOF–MS/MS)	[70]
34 components (heterophyllin A and B, alanine, lactate, lysine, taurine, sucrose, tyrosine, linolenic acid, γ-aminobutyrate, glutamine, raffinose, xylose etc.)	1H-NMR-based metabolomics coupled with HPLC	[47]
Free amino acid	NIR	[71]
Nucleosides and nucleobases	QTRAP LC–MS/MS	[55–57, 72]
Volatile components: palmitic acid (21.37%), 9,12-octadecadienoic acid ethylester (16.98%), trans-oleic acid (5.94%), chondrillasterol (3.99%), stigmast-7-en-3-ol (3.92%), 5,6-dihydroergosterol(2.48%), 1-monolinolein (2.35%)	GC–MS	[73]
Polysaccharide	High-performance size-exclusion chromatograph (HPSEC)	[74]
Water-soluble sugar	Phenol–sulfuric acid	[75]
Fingerprint	HPLC	[16, 37, 58–60, 76–78]
Fingerprint	GC–MS	[79]

### Analysis of cyclic peptides in *P. heterophylla*

Cyclic peptides are the characteristic components in *P. heterophylla*, and heterophyllin B (Fig. 1) is the most typical one. Its structure was elucidated as a cyclic octapeptide [cyclo-(Gly–Gly–Leu–Pro–Pro–Pro–Ile–Phe)] based on TLC, HPLC, MS and NMR analysis [40]. HSCCC (high speed counter current chromatography) was successfully applied for the separation of heterophyllin B from *P. heterophylla* [17]. Heterophyllin B was also used as quality control marker of *P. heterophylla* in Chinese Pharmacopoeia 2010 [41], but not in Chinese Pharmacopoeia 2015 [42]. This status indicated that heterophyllin B is not a reasonable marker for quality of *P. heterophylla.* Therefore, further research to find efficient markers for authenticity and quality evaluation of *P. heterophylla* is urgently needed.

Two cyclic peptides (heterophyllin A, B), 12 nucleosides, and 16 amino acids were simultaneously quantified by ultra-performance liquid chromatography tandem triple quadrupole mass spectrometry (UPLC-QQQ-MS/MS) [43]. The other studies focused on cyclic peptides were for simultaneous analysis of pseudostellarin A, C, D, and G using HPLC coupled with electrospray ionization tandem mass spectrometry (ESI–MSn) [25], and UPLC- quadrupole time of flight (QTOF)-MS/MS methods for analysis of pseudostellarin A, B, D, F, and heterophyllin A in *P. heterophylla* [44–46]. 1H-NMR-based metabolomics coupled with HPLC was also employed to investigate the metabolites in *P. heterophylla* [47], which has the unique advantages in the accurate identification of components.

### Analysis of carbohydrates in *P. heterophylla*

Polysaccharides are the main bioactive macromolecule components in *P. heterophylla*. Their pharmaceutical activities have been discussed in “Pharmaceutical activities of *P. heterophylla*” section. Polysaccharides in *P. heterophylla* were not well investigated to date, even if some of their beneficial effects such as antioxidant, immunostimulant and antitumor activities have been demonstrated. In fact, few types of polysaccharides have been identified in structure. However, the biological activities of polysaccharides are closely correlated to their molecular size, types and ratios of constituent monosaccharides, and features of glycosidic linkages (e.g., configuration and position of glycosidic linkages, and sequence of monosaccharides) [48, 49]. Recently, HPSEC, HPLC after 1-phenyl-3-methyl-5-pyrazolone (PMP) derivatization, NMR, Fourier transform infrared analysis (FT-IR) and chemical method including phenol–sulfuric acid, periodate oxidation, smith hydrolysis, methylation analysis, partial acid hydrolysis, have been applied for evaluating total contents, molecular sizes, types and ratios of constituent monosaccharides, and features of glycosidic linkages of polysaccharides in *P. heterophylla* [22, 26, 27, 50].

### Characterization of *P. heterophylla*

The isobaric tags for relative and absolute quantification (iTRAQ) MS/MS have been applied for discrimination of different habitats of *P. heterophylla* [51, 52]. Furthermore, Wu et al. developed a method based on Raman spectroscopy coupled with chemometric to discriminate the geographic regions of cultivation [12]. Near infrared (NIR) spectroscopy combined with support vector data description (SVDD) was attempted to identify the geographical origins of *P. heterophylla* [6]. NMR has also been developed for identification of wild *P. heterophylla* from different cultivated fields [47, 53]. In addition, high-throughput RNA sequencing (RNA-seq) was employed as de novo assembly for studying the transcriptome in *P. heterophylla*, and significantly differentially expressed genes in *P. heterophylla* from different fields were found [54].

Besides, nucleosides and nucleobases in *P. heterophylla* were quantified by QTRAP LC–MS/MS for evaluating the processing methods and discriminating different idioplasm resources of *P. heterophylla* [55–57]. HPLC and GC–MS fingerprints were also developed for identification of *P. heterophylla* [58–60].

## Conclusion

*Pseudostellaria heterophylla* is one of the well-known TCMs with multiple pharmacological activities in last decades. Even researchers evaluated the chemicals especially cyclic peptides in this plant, the methods for quality control of *P. heterophylla* are still not reasonable. Some chemicals such as oligosaccharides, which are rich in this plant based on our research (data will be published in others), were rarely evaluated. The investigation of oligosaccharides, with high amount in aqueous extract of *P. heterophylla*, may lead to develop a rational and scientific quality control methods for this herb.

## Data Availability

All reported or analyzed data in this review is extracted from published articles.

## Abbreviations

*P. heterophylla*: *Pseudostellaria heterophylla*
TCMs: traditional Chinese medicines
HSCCC: high-speed counter-current chromatography
HPLC: high performance liquid chromatography
TLC: thin layer chromatography
GC: gas chromatography
MALDI-MS: matrix-assisted laser desorption/ionization mass spectrometry
NIR: near infrared
NMR: nuclear magnetic resonance
BFs: bioconcentration factors
UPLC-QQQ-MS/MS: ultra-performance liquid chromatography tandem triple quadrupole mass spectrometry
ESI–MS: electrospray ionization tandem mass spectrometry
TOF: time of flight
PMP: 1-phenyl-3-methyl-5-pyrazolone
FT-IR: Fourier transform infrared analysis
iTRAQ: isobaric tags for relative and absolute quantification
SVDD: support vector data description
RNA-seq: RNA sequencing
